# Optimization of Electrically Active Magnetic Nanoparticles as Accurate and Efficient Microbial Extraction Tools

**DOI:** 10.3390/bios5010069

**Published:** 2015-02-05

**Authors:** Barbara C. Cloutier, Ashley K. Cloutier, Evangelyn C. Alocilja

**Affiliations:** 1 Large Animal Clinical Sciences, College of Veterinary Medicine, Michigan State University, 275 Slappy Drive, Hamilton, GA 31811, USA; 2 Department of Biosystems and Agricultural Engineering, Michigan State University, 115 Farrall Hall, East Lansing, MI 48824, USA; E-Mails: ak.cloutier@yahoo.com (A.K.C.); alocilja@msu.edu (E.C.A.)

**Keywords:** microbial pathogens, immune-magnetic separation, biosensor, magnetic nanoparticles

## Abstract

Food defense requires the means to efficiently screen large volumes of food for microbial pathogens. Even rapid detection methods often require lengthy enrichment steps, making them impractical for this application. There is a great need for rapid, sensitive, specific, and inexpensive methods for extracting and concentrating microbial pathogens from food. In this study, an immuno-magnetic separation (IMS) methodology was developed for *Escherichia coli* O157:H7, using electrically active magnetic nanoparticles (EAMNPs). The analytical specificity of the IMS method was evaluated against *Escherichia coli* O55:H7 and *Shigella boydii*, and was improved over previous protocols by the addition of sodium chloride during the conjugation of antibodies onto MNPs. The analytical sensitivity of the IMS method was greatest when a high concentration of antibodies (1.0 mg/mL) was present during conjugation. EAMNP concentrations of 1.0 and 0.5 mg/mL provided optimal analytical sensitivity and analytical specificity. The entire IMS procedure requires only 35 min, and antibody-conjugated MNPs show no decline in performance up to 149 days after conjugation. This analytically sensitive and specific extraction protocol has excellent longevity and shows promise as an effective extraction for multiple electrochemical biosensor applications.

## 1. Introduction

Food-borne microbial pathogens comprise one of the single largest threats to maintaining a safe food supply. The food defense (securing food sources against malicious biological attack) and food safety (identifying and eradicating contamination from natural sources) are growing increasingly relevant, as foods are processed and shipped further and faster than ever before [1,2]. Standard overnight culture methods for identifying microbial pathogens are no longer adequate, as the speed and breadth of food movement demands rapid, sensitive, specific, and economical means of extracting and detecting pathogens from food sources. Food and Drug Administration (FDA) inspections have dropped by 81% since 1972 and 47% between 2003 and 2006 [3]. Even with the new Food Safety Modernization Act (FSMA), the highest risk plants will only be inspected every three years by the FDA [4,5]. The FDA in the United States inspects less than 1% of the imported food supply before consumption and less than 0.2% of the imported food undergoes laboratory analysis at all [3]. Ultimately, companies are responsible for their own products and must protect their own brands. They cannot depend completely on government inspectors or third-party auditors to ensure the authenticity and safety of materials and products [2]. Decreasing the cost of a first line evaluation of food should allow a food company to test a greater percentage of their product, protecting their bottom line in preventing recalls and their brand reputation in the market. Moving the first line testing of food to the farm and field will allow both regulatory agencies and supply chain managers to find problems earlier before combination at the production or packing plant, benefiting both food safety and food defense.

The objective of this research was to develop an immuno-magnetic separation (IMS) methodology for food borne pathogens that is analytically sensitive and specific, highly inclusive and exclusive as well as inexpensive. Analytical sensitivity and specificity is the ability to isolate target cells with high efficiency, throughout the range of potential concentrations. Inclusivity and exclusivity are the ability to microbiologically discriminate against non-target cells, yet also include all versions of target cells. Maintaining an inexpensive cost element facilitates increasing the volume of food tested. IMS is a rapid method for extracting and concentrating a target analyte from its sample matrix. This is imperative due to the high level of interference the matrix of a food has on any diagnostic test [6]. IMS has been paired with a wide variety of biosensors for rapid detection of bacterial pathogens [2,7,8,9,10,11,12,13,14,15,16,17]. In IMS, micrometer or nanometer scale magnetic particles are immuno-functionalized with antibodies, incubated with the sample to bind target cells, and separated from the sample matrix through the application of a magnetic field. The magnetic particle-bound target can then be washed and concentrated, removing the matrix interference. The possibility of concentrating target cells prior to detection can eliminate the need for time-consuming pre-enrichment steps with a greater real time analytical sensitivity. In comparison to centrifugation, filtration, or capture of a target on an immuno-functionalized surface, IMS is simpler, and generally results in a higher capture efficiency due to the greater surface area available for target binding [7,18]. This is especially true of nano-sized particles. The surface chemistry of nano-sized particles such as surface tension, magnetization and sheer volume of surface area improve the amount of functionalized space for reaction to occur and thus improve the capture ability and longevity of the resultant IMS particles [7,18].

*Escherichia coli* O157:H7, a type of entero-hemorrhagic E. coli (EHEC), was chosen as the target strain for this study because it is a common and highly infective food- and water-borne pathogen, with a median infectious dose of 23 colony forming units (CFU) [19]. The standard method of identifying *E. coli* O157:H7 from unknown samples is through enrichment in selective media, followed by growth on differential agar. These are identified phenotypically, serologically, and toxigenically characterized by PCR, a process lasting several days. The standard method is able to detect <1 CFU/g in foods [20]. The IMS method presented here could be applied to extraction and concentration of *E. coli* O157:H7 from food samples, eliminating the standard method’s overnight enrichment step. By pairing IMS with PCR or nearly any other rapid detection method, negative or presumptive positive results could be obtained in a few hours or less. The development and application of electrically active magnetic nanoparticles (EAMNPs) for IMS has been previously reported by this laboratory [21,22]. The EAMNPs consist of an iron oxide core with a polyaniline coating, which enables them to not only extract target cells, but also to function as the signal transducer in certain electrical detection platforms. The reported method was effective in isolating target cells from pure culture and food matrices with reasonable analytical sensitivity, but when challenged with non-target organisms, it demonstrated inadequate analytical specificity. Adjustment of the environment of the MNPs during conjugation of the antibody probe was hypothesized to correct this problem. Some results from this portion of the study have been published in the International Journal of Food Safety, Nutrition and Public Health—Food Defense edition [23].

## 2. Experimental Section

To optimize the use of EAMNPs to extract and concentrate microbial targets, the following hypothesis was proposed: Monoclonal antibody conjugated-EAMNPs (Mab-EAMNPs) can selectively extract and concentrate 1.0 to 1.0 × 10^9^ CFU/mL of *E. coli* O157:H7 in broth samples. This hypothesis was subdivided into four distinct sub-hypotheses. These sub-hypotheses were developed using the previously reported methodology as a starting point with the goal of developing a new IMS methodology for *E. coli* O157:H7 that has both analytical sensitivity and specificity [21,22]. It was hypothesized that the analytical sensitivity and specificity of the IMS methodology is affected by:

(a) The addition of sodium chloride to a concentration of about 0.14 M during conjugation of antibodies onto MNPs.
(b) The concentration of antibodies present during conjugation of antibodies onto MNPs.
(c) The concentration of Mab-EAMNPs present during IMS.
(d) The number of days elapsed since conjugation of antibodies onto EAMNPs, which affect the capture evaluation by culture after IMS.

In order to test the four hypotheses stated above, five factors (sodium chloride addition, antibody concentration, Mab-EAMNP concentration, and the age of the Mab-EAMNP solution) were evaluated in terms of their effects on the analytical sensitivity and specificity of the proposed IMS methodology. Therefore, every experiment was applied to three different bacterial species individually: *E. coli* O157:H7 (target species), *E. coli* O55:H7, and *Shigella boydii* (both non-target species). *E. coli* O55:H7 is another EHEC serotype closely related to *E. coli* O157:H7. *S. boydii* bears less genotypic and phenotypic similarity to the target organism, but it is a commonly encountered foodborne pathogen and also produces shiga-toxin like *E. coli* O157:H7. The non-target organisms chosen for this study correspond with the recommendations made by the AOAC Task Force on Best Practices in Microbiological Methodology [24].

To test Hypothesis 1a, Mab-EAMNPs made with the addition of sodium chloride were compared to those made without sodium chloride. (In either case, the initial concentration of antibodies was 1.0 mg/mL). Both with and without sodium chloride, three concentrations (1.0 mg/mL, 0.5 mg/mL, and 0.1 mg/mL) of Mab-EAMNP were used to perform IMS.

To test Hypothesis 1b, Mab-EAMNPs made with an initial antibody concentration of 1.0 mg/mL were compared to those made with an initial antibody concentration of 0.5 mg/mL. In either case, sodium chloride was added during conjugation. With both 1.0 mg/mL of antibodies and 0.5 mg/mL of antibodies, three concentrations (1.0 mg/mL, 0.5 mg/mL, and 0.1 mg/mL) of Mab-EAMNP were used to perform IMS.

To test Hypothesis 1c, Mab-EAMNPs were made with the addition of sodium chloride and with an initial antibody concentration of 1.0 mg/mL. Each of the four concentrations (1.5 mg/mL, 1.0 mg/mL, 0.5 mg/mL, and 0.1 mg/mL) of Mab-MNPs was used to perform IMS.

To test Hypothesis 1d, Mab-EAMNPs were made with the addition of sodium chloride, and with initial antibody concentrations of both 1.0 mg/mL and 0.5 mg/mL. Two concentrations (1.0 mg/mL and 0.5 mg/mL) of Mab-EAMNPs were used to perform IMS at various points from 0 to 150 days after conjugation.

## 3. Materials and Methods

*E. coli* O157:H7 strains, *E. coli* non H7 strains and non *E. coli* bacterial strains were obtained from the Shiga toxin-producing *E. coli* (STEC) Center collection at Michigan State University (MSU) (Shannon Manning, MPH, PhD), the Nano-Biosensors Laboratory at MSU (Evangelyn Alocilja, PhD), Neogen Inc. Research and Development, Lansing, Michigan (Jennifer Rice, DVM, PhD) and the University of Georgia, Center for Food Safety (Dr. Michael Doyle, PhD). From frozen purified culture stocks (stored at −80 °C), colonies were isolated by streak-plate method on trypticase soy agar (BD Biosciences, MD) plates. A single colony was used to inoculate a vial of tryptic soy broth (BD Biosciences, MD) and grown overnight at 37 °C. A 1 mL aliquot of the liquid culture was transferred to a new vial of broth and stored at 37 °C for up to 6 days. This culture was used to inoculate a new vial of broth with 1 mL of inoculum 10 to 24 h before each experiment to produce fresh bacterial cells which were serially diluted in 0.1% (w/v) peptone water (Fluka-Biochemika, Switzerland) prior to their use in the IMS procedure. Viable cells were enumerated by microbial plating on MacConkey agar with sorbitol (SMAC) (BD Biosciences, MD or Neogen Inc., MI), according to standard rules for plate counting [20]. Optical Density at 600 nanometers (OD 600) spectrophotometer readings (BIO-RAD Smartspec 3000, Hercules, CA, USA) were taken from each culture before use, as compared to blank Trypticase Soy Broth (TSB). Three readings were taken and averaged together.

### 3.1. EAMNP Production

Ferric chloride hexahydrate (EMD Chemicals, Bedford, MA, USA), sodium acetate (CCI Chemicals, Vernon, CA, USA), sodium acrylate, sodium chloride (NaCl), ethylene glycol, ethylenediamine, hydrochloric acid, aniline, iron (III) oxide nanopowder, ammonium persulfate, methanol, and diethyl ether were used as received from Sigma Aldrich (St. Louis, MO, USA) in the synthesis of the EAMNPs. EAMNPs were synthesized by polymerization and acid doping of aniline monomer around gamma iron (III) oxide (γ-Fe_2_O_3_) nanoparticles, using a slightly modified published procedure [21]. Briefly, 0.650 g of iron (III) oxide nanopowder were dispersed in 50 mL of 1 M HCl, 10 mL of deionized water and 0.4 mL of aniline monomer by sonication in an ice bath for 1 h. A volume of 20 mL of 0.2 M ammonium persulfate (as oxidant) was added drop-wise to the above solution under continuous magnetic stirring. Color change from rust brown to dark green indicated formation of electrically-active (green) polyaniline over the smaller (brown) γ-Fe_2_O_3_ nano-particles. The solution was stirred for 2 h in an ice bath and was filtered through a qualitative grade filter (2.5 µm pore size, Ahlstrom, grade 601). The supernatant thus obtained was successively filtered through a nitrocellulose membrane filter (1.2 µm pore size, Millipore) followed by washings with 10 mL each of 1M HCl, 10% (v/v) methanol, and diethyl ether. The particles were dried overnight at room temperature under vacuum. The particles ranged in size from 1.2 to 2.5 µm and displayed a room temperature saturation magnetization of 30 emu/g.

### 3.2. EAMNP Antibody Conjugation

Nanoparticles were immune-functionalized with monoclonal anti-*E. coli* O157:H7 antibodies obtained from Meridian Life Science, Inc. (Saco, ME, USA). Polysorbate-20 (Tween-20), Triton X-100, phosphate buffered saline (PBS), Trizma base, casein, and sodium phosphate (dibasic and monobasic) were used in the IMS procedure. All of the above reagents, unless otherwise noted, were purchased from Sigma-Aldrich (St. Louis, MO, USA). All solutions and buffers used in this study were prepared in deionized (DI) water (from Millipore Direct-Q system) as follows: PBS buffer (10 mM PBS, pH 7.4), wash buffer (10 mM PBS, pH 7.4, with 0.05% Tween-20 or 0.05% Triton-X100), phosphate buffer (100 mM sodium phosphate, pH 7.4), and blocking buffer (100 mM Tris–HCl buffer, pH 7.6, with 0.01% w/v casein). Magnetic separations were performed with a commercial magnetic separator (Promega Corporation, Madison, WI, USA). Hybridization of biological materials was carried out at room temperature with rotation on a tube rotisserie (Labquake, Thermo Scientific, MA, USA). Scanning electron micrographs were acquired using field-emission scanning electron microscopy (JOEL 7500F, acceleration voltage of 5 kV). A superconducting quantum interference device magnetometer (Quantum design MPMS SQUID) was used for magnetic characterization of EAMNPs. Mab-conjugation of the EAMNPs was carried out by physical adsorption of antibodies onto the polyaniline surface. Electrostatic interactions between the negatively charged constant (Fc) portion of the antibodies and the positively charged polyaniline surface are thought to play a role in adsorption and orientation of the biomolecules onto the EAMNPs [22]. Successful conjugation of antibodies onto EAMNPs was confirmed by measuring the quantity of antibody in the post-hybridization supernatant with a commercial fluorescence-based protein quantification kit. The measured protein concentration in the supernatant was significantly lower than the concentration of antibodies initially added to the MNPs (data not shown), indicating that antibodies were retained on the MNPs during hybridization.

EAMNPs were conjugated with monoclonal antibodies at an initial EAMNP concentration of 10 mg/mL (1% solid). Two different initial concentrations of monoclonal antibodies were used during conjugation: 1.0 and 0.5 mg/mL. The conjugation of antibodies onto EAMNPs was performed both with and without the addition of sodium chloride. A 100 µL aliquot of monoclonal, anti-*E. coli* O157:H7 antibody (suspended in 0.1 M phosphate buffer) was added to EAMNPs suspended in PBS, yielding a final antibody concentration of either 1.0 or 0.5 mg/mL. The mixture was hybridized on a rotisserie-style rotator for 1 h at room temperature, with 25 µL of 10X PBS being added after the first 5 min of hybridization, to increase the sodium chloride content of the suspension to approximately 0.14 M. (For select experiments, the 10 × PBS was omitted). Following hybridization, the EAMNP-antibody conjugate was magnetically separated, the supernatant removed, and the conjugate re-suspended in 250 µL of blocking buffer (0.1 M tris buffer with 0.01% casein) for 5 min. Again the conjugate was magnetically separated, the supernatant removed, and the conjugate re-suspended in 250 µL of blocking buffer, this time for 1 h with rotation. Finally, the EAMNP-antibody conjugate was magnetically separated, the supernatant removed, and the conjugate re-suspended in 2.5 mL of 0.1 M phosphate buffered saline (PBS). The final concentration of EAMNPs in each solution was 1.0 mg/mL. Immuno-conjugated EAMNPs (Mab-EAMNPs) were stored at 4 °C. Prior to experimental use, Mab-EAMNPs were either magnetically separated and concentrated or further diluted in 0.1 M PBS, in order to obtain solutions of Mab-EAMNPs at the following concentrations: 1.5 mg/mL, 1.0 mg/mL, 0.5 mg/mL, and 0.1 mg/mL EAMNPs.

### 3.3. Immuno-Magnetic Separation (IMS) and Plating of Bacteria

**Figure 1:**
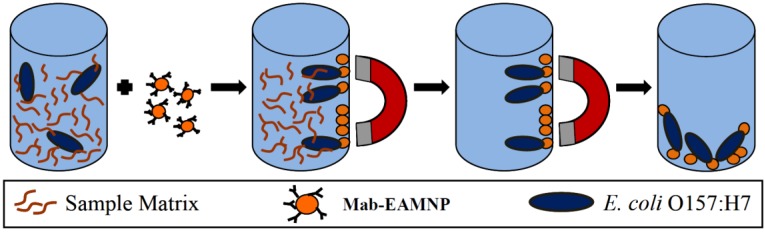
Immuno-magnetic separation procedure (IMS): sample plus Mab-EAMNPs → magnetic separation of target cells → removal of sample matrix → purified *E. coli* O157:H7-Mab-EAMNP complexes.

Serial dilutions of each bacterium were independently prepared in 0.1% (w/v) peptone water, along with subsequent negative, positive and blank controls [25,26,27,28,29,30,31]. The standard positive control used was Spinach pGFPuv. Three or four of the pure dilutions of each bacteria were plated (100-mL aliquots) on sorbitol MacConkey agar (SMAC) and incubated at 37 °C overnight. For IMS, 50 mL of Mab-EAMNPs and 50 mL of the appropriate bacterial dilution were combined with 400 mL of 0.01 M PBS (pH 7.4), and hybridized with rotation at room temperature for 30 min. After hybridization, the cell-Mab-EAMNP complexes were magnetically separated and the supernatant removed. Complexes were washed twice in wash buffer (0.01 M PBS containing 0.05% Triton-X100) and finally re-suspended in 0.5 mL of 0.01 M PBS. The IMS procedure required 40 min, and is depicted in Figure 1.

A 100-mL aliquot was placed on SMAC and incubated at 37 °C overnight. The number of colony-forming units (CFU) in the 100-mL aliquot was determined by manually counting the colonies on each plate. For every experimental case (*i.e.*, particular combination of Mab-EAMNP concentration, and bacteria), a minimum of two bacterial dilutions underwent IMS and were plated. In most cases, a full spectrum of dilutions from 10^−1^ to 10^−9^ were run as independent units. For the lower dilutions from 10^−1^ to 10^−5^, the final IMS solution was diluted from 5 times to 1 time, respectively, to obtain countable plates. For dilutions from the 10^−8^ and 10^−9^ series, all 500 µL present were plated.

Calculation of bacterial cell concentrations in both pure and IMS separated samples were carried out according to rules provided by the United States Food and Drug Administration’s Bacteriological Analytical Manual [20,28]. Plate counts between 25 and 250 colonies were used to calculate what the *original* cell concentrations were in CFU/mL. If all plate counts for a given case fell outside of this range, estimates were made according to FDA rules. Any plate count of zero was therefore estimated using Equation (1).

1.0 × d = CFU/mL   d = dilution factor plated
(1)

This leads to an increased estimate of the captured values for those samples where nothing grew and greatly increases the perceived capture of the negative control organisms. This was done to facilitate the statistical analysis comparing the capture of the three organisms and avoid zero measurements. This should not be considered the analytical specificity or analytical sensitivity limits of the extraction.

### 3.4. Statistical Analysis

The calculated concentrations of cells captured by IMS (in CFU/mL) were converted to their log10 values. The log10 conversion also normalizes the distribution [31,32]. Statistical analysis was performed using SPSS software (Armonk, NY, USA). Missing values were computed with hot-deck imputation or excluded analysis by analysis. Independent, two-tailed T-tests were used to compare experiments in which sodium chloride was added during conjugation, to experiments in which sodium chloride was omitted. Similarly, experiments in which the antibody concentration was 1.0 mg/mL were compared to experiments in which the antibody concentration was 0.5 mg/mL. All experimental results were included for these two analyses. The results presented were not controlled for initial bacterial culture composition and all calculations were estimated if no growth occurred.

Subsequent analysis was performed using both one-way ANOVA and independent two-tailed T-tests, to evaluate the effect of Mab-EAMNP concentration. This analysis included only the results of experiments, which had the 1.0 mg/mL antibody concentration and the addition of sodium chloride during conjugation. (In the previous analyses, these parameters were statistically determined to result in better overall IMS performance). Analyses which showed abnormal data distributions were re-evaluated with Kruskal-Wallis or Mann-Whitney U tests as needed. The results presented were not controlled for initial bacterial concentration off the growth curves. All analyses were calculated with 95% confidence intervals (α = 0.05).

## 4. Results and Discussion

**Figure 2:**
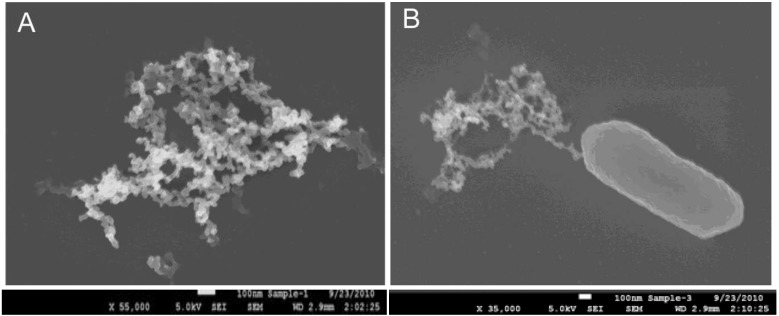
Scanning electron micrograph of (**a**) an individual EAMNP with diameter of approximately 1.3 µm; and (**b**) Mab-EAMNP bound to an *E. coli* O157:H7 cell.

Immuno-magnetic capture of *E. coli* O157:H7 cells were quantified by plate counts , but capture was also visually confirmed by scanning electron microscopy (SEM). Figure 2 shows SEM images of (a) an individual EAMNP with diameter of approximately 1.3 µm; and (b) a Mab-EAMNP bound to an *E. coli* O157:H7 cell, after washing twice to remove non-specifically bound cells.

### 4.1. Hypothesis 1a: Effect of Sodium Chloride Addition during Conjugation

**Figure 3:**
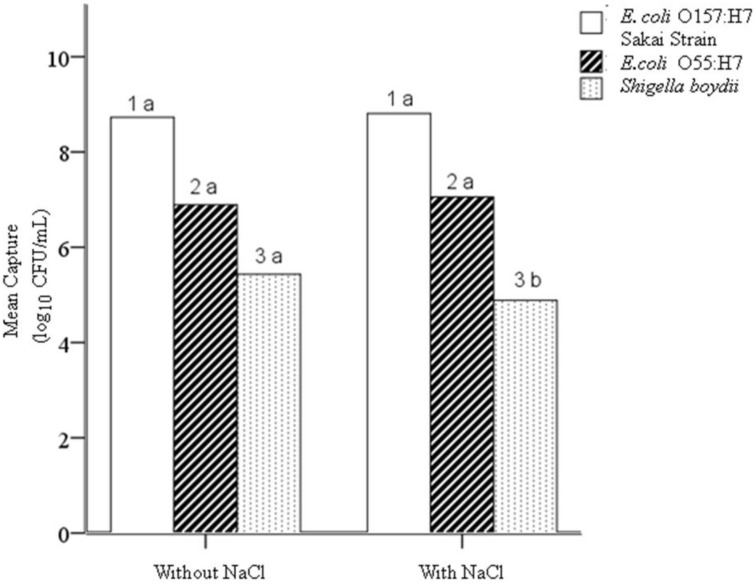
Mean concentration (log_10_ of CFU/mL) of each bacterial culture captured in IMS, using Mab-EAMNPs made with and without the addition of sodium chloride. Statistical comparisons were made within numbered groups (1–3), and letters (a or b) indicate significant differences (α = 0.05, n = 178; p = 0.029). Zero counts were estimated to facilitate analysis [23].

Two-tailed independent T-tests performed on the mean concentrations of captured cells (log10 of CFU/mL) for all three bacteria in the initial study showed that the addition of sodium chloride (compared with omitting sodium chloride) causes a significant decrease in capture of the negative control *S. boydii* (*n* = 178; *p* = 0.029), with no significant effect on the capture of the target *E. coli* O157:H7 or the other negative control *E. coli* O55:H7. The addition of 0.14 M sodium chloride during conjugation of antibodies onto EAMNPs increases the specificity at all Mab-EAMNP concentrations evaluated, and has no effect on sensitivity (Figure 3).

### 4.2. Hypothesis 1b: Effect of Antibody Concentration during Conjugation

**Figure 4:**
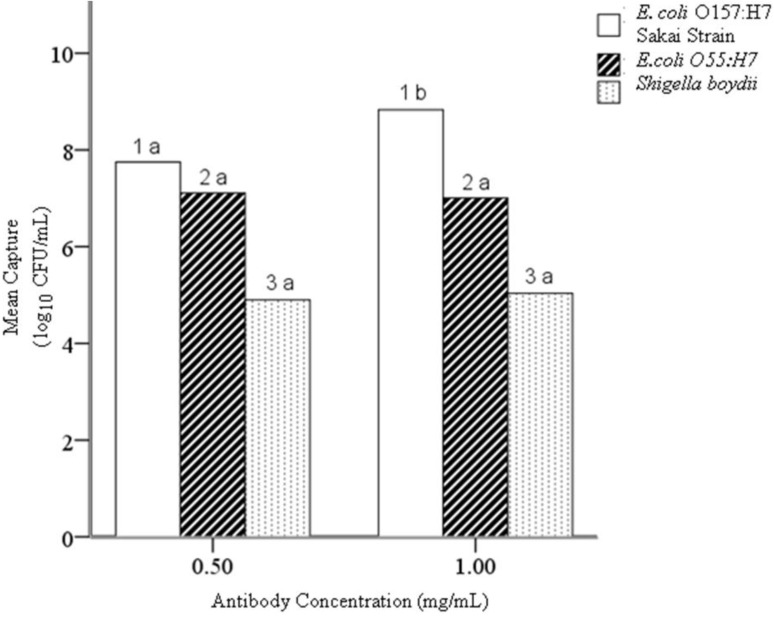
Mean concentration (log_10_ of CFU/mL) of each bacterial culture captured in IMS, using Mab-EAMNPs made with 1.0 mg/mL antibody and with 0.5 mg/mL antibody. Statistical comparisons were made within numbered groups (1–3), and letters (a or b) indicate significant differences (α = 0.05, *n* =178, *p* = 0.018). Zero counts were estimated to facilitate analysis [23].

Two-tailed independent T-tests performed on the mean concentrations of captured cells (log_10_ of CFU/mL) for all three bacteria showed that the higher antibody concentration (1.0 mg/mL) caused a significant increase in capture of the target *E. coli* O157:H7 (*n* = 178; *p* = 0.018), with no significant effect on the capture of the negative control microorganisms. The higher antibody concentration (1.0 mg/mL) during conjugation increases the analytical sensitivity of EAMNPs at all Mab-EAMNP concentrations evaluated, and has no effect on analytical specificity (Figure 4).

### 4.3. Hypothesis 1c: Effect of Mab-EAMNP Concentration during IMS

**Figure 5:**
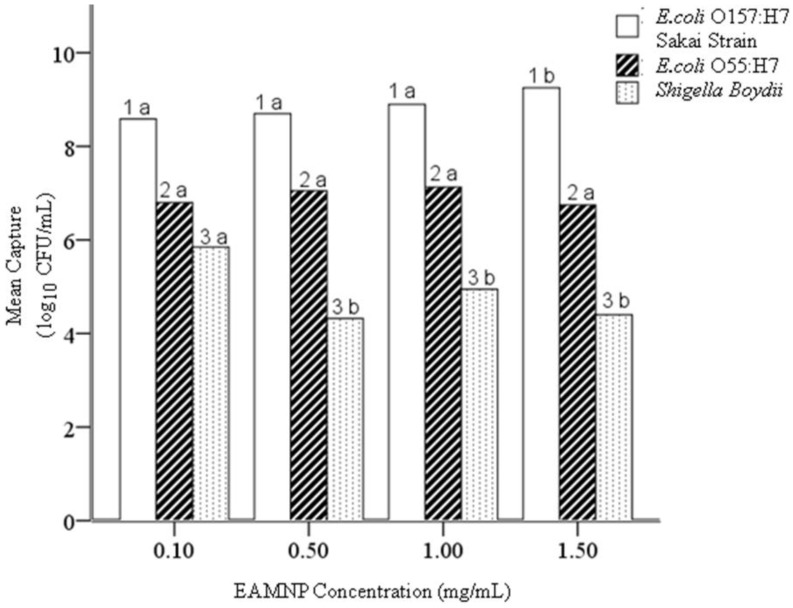
Mean concentration (log_10_ of CFU/mL) of each bacterial culture captured in IMS, using Mab-EAMNPs, at concentrations of 1.5 mg/mL, 1.0 mg/mL, 0.5 mg/mL, and 0.1 mg/mL. Statistical comparisons were made within numbered groups (1–3), and letters (a or b) indicate significant differences (α = 0.05). Zero counts were estimated to facilitate analysis [23].

One-way ANOVA was performed on the mean concentrations of captured cells (log_10_ of CFU/mL) for all three bacteria, separated according to Mab-EAMNP concentration [31]. No significant difference in the capture of the target *E. coli* O157:H7 Sakai was observed at any Mab-EAMNP concentration with this test (LDS and Bonferroni pairwise comparison). However, the ANOVA homogeneity of variance test showed non-normal distributions for various bacteria. To account for the non-normality observed in the ANOVA, independent T-tests were also performed for all three bacteria, and these did show some significant differences in medians, with the nonparametric comparison (using the Kruskal-Wallis test for median and distribution, or the Mann-Whitney two-sample comparison). From these statistical analyses, the following conclusions were drawn:

EAMNPs at both 1.5 and 0.1 mg/mL are less analytically specific than EAMNPs at either 1.0 or 0.5 mg/mL. Despite the small number of data points (*n* = 5) for EAMNPs at 1.5 mg/mL, this concentration is more analytically sensitive than any other concentration of EAMNPs. Based on these statistical results, null Hypothesis 1c is rejected. The concentration of Mab-EAMNPs present during IMS has an effect on both analytical sensitivity and specificity (Figure 5). In most cases where the Mab-EAMNP concentration had a significant effect on bacterial capture, concentrations of 1.0 and 0.5 mg/mL provide the optimal analytical sensitivity and specificity.

### 4.4. Hypothesis 1d: Effect of Age of Mab-EAMNP

#### Solution during IMS

**Figure 6:**
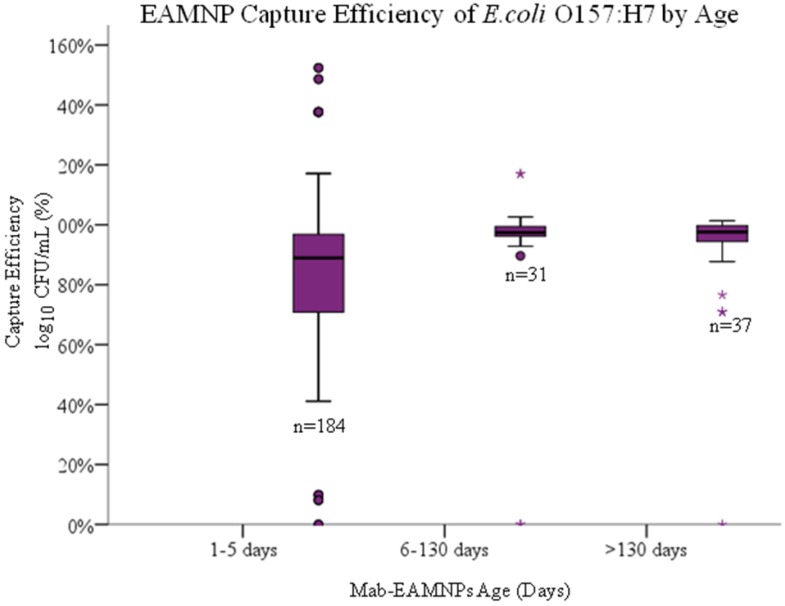
Capture efficiency (log_10_ of CFU/mL captured/ log_10_ of CFU/mL present) of each *E. coli* O157:H7 sakai strain captured in IMS, using Mab-EAMNPs, at varying days from conjugation stored at refrigerated temperatures from (1–5 days, *n* = 184; 6–130 days, *n* = 31; 130 to 149 days, *n* = 37). There was no statistical difference between any of the three groups.

Longevity of the Mab-EAMNP solutions was also evaluated by one-way ANOVA and independent two-tailed T-tests. With the previously reported method of conjugating antibodies onto EAMNPs [21,22], long-term storage of Mab-EAMNP solutions (at 4 °C) resulted in poorer IMS performance. This observation led to Hypothesis 1d, that the number of days elapsed since conjugation of antibodies onto EAMNPs will affect the analytical sensitivity and specificity of IMS. One-way ANOVA and independent two-tailed T-tests were performed on the mean concentration of captured cells (log10 of CFU/mL) for all three bacteria, comparing the experimental results obtained from Mab-EAMNP solutions ranging in age from 0 to 149 days (Figure 6). Regardless of which statistical test was applied, no significant difference was observed in IMS capture of any of the three bacteria. Based on these statistical results, null Hypothesis 1d is retained. Days elapsed since conjugation of antibodies onto MNPs (stored at 4 °C), from 0 to 149 days, has no effect on analytical sensitivity or specificity.

By changing several portions of the conjugation step for the EAMNPs, the new IMS methodology reported here was able to isolate *E. coli* O157:H7 Sakai strain with excellent analytical sensitivity, and discriminates against *E. coli* O55:H7 and *Shigella boydi.* Additionally, this methodology requires a smaller volume of EAMNPs per extraction, and results in an EAMNP-antibody conjugate with a much longer storage life, as compared to our previous method. Both of these improvements contribute to a lower overall cost of the IMS assay. Over fifty different independent runs in duplicate or triplicate were accomplished at all concentrations from 1–2 CFU/mL to 1.0 × 10^10^ CFU/mL.

Conjugation of antibodies onto EAMNPs was carried out in phosphate buffer at pH 7.4. A slightly basic pH such as this is recommended for optimal adsorption of the Fc (constant) portion of the antibody [33], which positions the Fab (antigen-binding) portion outward for maximum target-binding capacity. It has also been reported that the addition of sodium chloride at or near physiological concentration (about 0.15 M) increases adsorption efficiency of antibodies onto microspheres [25,29,34,35]. This was the foundation for Hypothesis 1a, that the addition of sodium chloride during conjugation will affect the analytical sensitivity and specificity of IMS. Its improvement is possibly due to the more physiologic conditions mimicking the antibody’s primary functional environment. The addition of sodium chloride during conjugation is a simple and inexpensive procedural change able to enhance IMS performance for any application.

During the conjugation of antibodies onto EAMNPs, EAMNPs were present at a concentration of 10 mg/mL, or 1% solids. The solution volume was kept small (250 µL, until post-conjugation dilution) in order to increase the speed and frequency of interactions between antibodies and EAMNPs during conjugation. Monoclonal antibodies were added at relatively high concentrations of 1.0 or 0.5 mg/mL during conjugation. Bangs Laboratories recommends 3–10 times the antibody concentration needed to create a monolayer (calculated amount) to ensure favorable stoichiometry for helping the Fc region to adsorb first [33]. This was the foundation for Hypothesis 1b, that the concentration of antibodies present during conjugation will affect the analytical sensitivity and specificity of IMS. The antibody concentration changes in the conjugation protocol did have a statistically significant improvement in the analytical sensitivity of the extraction as determined by culture (Figure 3). This may be due to competition. The more antibodies present, the more likely the resultant orientation of the antibody is Fc portion down since it is the smallest in diameter. Although consumption of more antibodies increases the cost of the assay, it is worthwhile for some IMS applications. Since the infectious dose of *E. coli* O157:H7 has a median of 23 CFU, high analytical sensitivity is a critical feature in any IMS assay for this organism [19]. However, if IMS is being applied to a pathogen like *Bacillus cereus*, with an infectious dose greater than 1.0 × 10^6^ *cells* [20], then decreasing the cost of the assay would likely be of greater value than increasing the analytical sensitivity, and a lower antibody concentration may be ideal.

With the objective of developing an IMS methodology that is analytically sensitive and specific, but also practical and cost-effective, the concentration of Mab-EAMNPs employed in IMS was identified as an important parameter to be optimized. This concern led to Hypothesis 1c, that the concentration of Mab-EAMNPs present during IMS will affect the analytical sensitivity and specificity of IMS. The concentration of Mab-EAMNPs present during IMS had a significant effect on bacterial capture, concentrations of 1.0 and 0.5 mg/mL provide the optimal analytical sensitivity and specificity. These findings offer the experimenter some flexibility in tailoring the IMS methodology to suit a particular application, depending on whether analytical sensitivity or specificity is of greater concern. A very low Mab-EAMNP concentration (such as 0.1 mg/mL) could also be employed to drastically decrease the cost of the assay in cases where neither analytical sensitivity nor specificity must be optimal (for example, high-throughput yes/no screening of food products, with tolerance levels greater than zero).

The EAMNPs were shown to have excellent longevity, with no decline in performance up to 149 days after conjugation. This data includes data taken with no control over the age of the culture used as a testing solution. This provides the operator much more flexibility in reaction time if the conjugate can be made ahead and used when needed. It also allows the resultant biosensor evaluation of time to result to exclude the time needed to conjugate the EAMNPs and only measure the time for the actual IMS.

## 5. Conclusions

This cumulative total of 450 repetitive broth challenges yielded statistically significant extraction and culture detection at all concentrations, with appropriate negative, positive and blank controls included. The entire IMS procedure requires only 35 min. The experiments designed and executed in this study provided conclusive results, allowing the initial hypotheses to be either rejected or retained. The concentration of MAb-EAMNP during use can be decreased compared to commercially available IMS methods and initially reported methods using this IMS without significant changes to reported analytical sensitivity and specificity. Limitations of this extraction method include the fact that both viable and non-viable cells are extracted with this methodology. Further studies are designed and being implemented to evaluate the Mab-EAMNP to determine the reaction kinetics of non-viable verses viable cells on the antibody target region in broth cultures. Limits of detection, inclusivity and exclusivity of microbial families and biosensor platform experiments are necessary before validation trials of the whole biosensor can proceed. The ultimate goal of this extraction is to be able to multiplex many EAMNPs with different Mab targets to allow multiplexing. Future multiplexing with multiple EAMNP and multiple bacterial targets could have interactions between the EAMNPs or between the mixed antibodies. Certain matrices may remove the Mab from the surface of the EAMNPs and make their use in that matrix impossible. The largest drawback to this method is the need for refrigeration of the Mab-EAMNPs. When field based technologies are discussed, shelf stable reagents are an advantage.
